# Huntingtin Fragments and SOD1 Mutants Form Soluble Oligomers in the Cell

**DOI:** 10.1371/journal.pone.0040329

**Published:** 2012-06-29

**Authors:** Yang-Nim Park, Xiaohong Zhao, Mark Norton, J. Paul Taylor, Evan Eisenberg, Lois E. Greene

**Affiliations:** 1 Laboratory of Cell Biology, National Heart Lung Blood Institute, National Institutes of Health, Bethesda, Maryland, United States of America; 2 St. Jude Children's Research Hospital, Memphis, Tennessee, United States of America; University Medical Center Groningen, University of Groningen, The Netherlands

## Abstract

Diffusion coefficients of huntingtin (Htt) fragments and SOD1 mutants expressed in cells were measured using fluorescence correlation spectroscopy. The diffusion mobilities of both non-pathological Htt fragments (25 polyQs) and pathological Htt fragments (103 polyQs) were much slower than expected for monomers suggesting that they oligomerize. The mobility of these fragments was unaffected by duration of expression or by over-expression of Hsp70 and Hsp40. However in cells with HttQ103 inclusions, diffusion measurements showed that the residual cytosolic HttQ103 was monomeric. These results suggest that both non-pathological and pathological Htt fragments form soluble oligomers in the cytosol with the properties of the oligomers determining whether they cause pathology. SOD1 with point mutations (A4V, G37R, and G85R) also had slower diffusional mobility than the wild-type protein whose mobility was consistent with that of a dimer. However, the decrease in mobility of the different SOD1 mutantsdid not correlate with their known pathology. Therefore, while soluble oligomers always seem to be present under conditions where cell pathology occurs, the presence of the oligomers, in itself, does not determine the extent of neuropathology.

## Introduction

There are a large number of neurodegenerative diseases that are caused by the presence of misfolded proteins, including Alzheimer's disease, Parkinson's disease, Huntington's disease, prion disease, and motor neuron disease. In all of these diseases, the aggregated misfolded protein forms abnormal deposits in the neurons. Many of these neurodegenerative diseases are caused by mutations in the proteinsthemselves, as with SOD1, alpha-synuclein, and the polyglutamine(polyQ) family of inherited neurological diseases, the most prominent of which is Huntington's disease. In the polyQ family of diseases, pathology is caused by an expansion of the polyQ repeat region of the protein; both the age of onset and the severity of the neurodegenerative disease is dependent on the length of the polyQ repeat region [1].

Studies examining the aggregation properties of the polyQ family of proteins have ranged from biophysical characterization of aggregates using pure proteins to analysis of these proteins in tissue culture and animal models. In Huntington's disease, cleavage of the full-length 348-kDa huntingtin protein (Htt) is essential for its pathogenicity [2] and expression of the N-terminal fragments of Htt, which contain the polyQ repeat region, causes toxicity in cell and animal models [3]. However, there are conflicting data as to the conformational state of the Htt fragments that cause this toxicity. Several studies have suggested that the aggregate, itself, is toxic since there is a correlation between aggregate formation and toxicity in tissue culture models [4], [5], [6]. Paradoxically, however, by following individual cells with and without aggregates over long periods of time, several research groups found that cells with aggregates were actually more likely to survive [7], [8]; rather than the aggregate itself, the amount of diffuse intracellular Htt fragment was found to correlate with cell death [7], [9]. As for the nature of the diffuse toxic species, monomers and soluble oligomers of Htt fragments with expanded polyQ repeats have been reported to be cytotoxic [8], [9], [10]. Soluble oligomers of Htt with expanded polyQ repeat regions have been observed by many different techniques in studies using purified proteins, lysates from tissues expressing Htt fragments, cells expressing Htt fragments, and brains from mouse models of Huntington's disease [8], [11], [12], [13], [14].

Similar to the polyQ family of proteins, superoxide dismutase 1 (SOD1) is another protein whose aggregation is correlated with a neurodegenerative disease, in this case familial amyotrophic lateral sclerosis (FALS). SOD1, a copper-zinc binding enzyme that functions as an antioxidant, forms a homodimer composed of 16-kDa subunits. Aggregation of SOD1 is caused by a various single point mutations, of which more than 100 have been identified. Interestingly, these mutations are scattered throughout the protein affecting a variety of different properties such as its dimerization, net charge, or affinity for metals, but the only consistent consequence of all these mutations is promotion of aggregation [15]. As in the case of the polyQ proteins, the role of mutant SOD1 aggregates in causing neurodegeneration is not clear, especially since SOD1 aggregates are formed relatively late during progression of the disease [16]. Although aggregates of mutant SOD1 are clearly associated with FALS, it has been suggested that soluble oligomers may initiate the disease with larger aggregates implicated in rapidly progressing events in the final stages of the disease [17].

Aggregation properties of fluorescently-labeled polyQ proteins have been studied in cells using fluorescence resonance energy transfer (FRET). Energy transfer was found to occur in cells expressing polyQ81, but not polyQ19 [11], which suggests that only the former protein formed oligomers. On the other hand, another study found that both shorter non-pathological and longer pathological length Htt fragments gave FRET positive signals [8], although the percentage of FRET positive cells and the FRET efficiency were significantly reduced in cells expressing the non-pathological Htt fragments. Therefore, although these two FRET studies indicate that pathological polyQ fragments with expanded polyQ repeat regions form soluble oligomers in the cytosol, they disagree as to whether non-pathological fragments with short polyQ repeat regions form soluble oligomers. A possible cause for the difference between these studies might be that they used different polyQ constructs were used in these studies. In addition, FRET signals have the technical drawback that both the abundance of the acceptor molecules and the stoichiometry of the donor and acceptor molecules in a complex affect the signal [18].

As for mutants of SOD1, they too form visible aggregates, but these aggregates are considerably more porous than Htt aggregates [19]. Moreover, in contrast to Httfragments, in cells expressing point mutants of SOD1 (G85R or G93A), no FRET signalswere detected between mutant SOD1 molecules in the cytosol and even in aggregates [19]. Oligomers of SOD1 have not yet been detected incells, but the presence of soluble oligomers of recombinant wild-type and mutant SOD1 in the metal free form have been shown to occur in solution under physiological conditions [20], [21], [22].

Another fluorescence technique that has been used to detect microscopic aggregates is fluorescence correlation spectroscopy (FCS), which measures the diffusion coefficients of proteins. This technique has been used to detect aggregates of purified AlexaFluor488-labeled polyQ peptides in solution and GFP-labeled polyQproteins in lysates from transfected cells [11], [23], but it has not yet been applied directly to tissue culture cells. In the present study, we used FCS microscopy to detect aggregates in the cytosol of cells expressing either Htt fragments of various sizes, both pathologic and non-pathologic, or SOD1 mutants. Both non-pathologic and pathologic Htt fragments have diffusional mobilities much slower than that of monomer, indicating that these proteins form small soluble oligomers in the cell cytosol. The diffusional mobilities of SOD1 with single point mutations (A4V, G35R or G85R) also indicatedthe presence of oligomers since the mutantsdiffused slower than a dimer, the normal state of the wild-type protein. However, the extent of oligomerization did not correlate with the pathological effects of these different SOD1 mutants. Therefore, although both Htt and SOD1 oligomers are present under conditions where cell pathology occurs, our results suggest that the presence of oligomers is just one of the factors that determinewhether pathology occurs and how severe it is.

## Materials and Methods

### Constructs

Fluorescent Htt constructs used in this study were described previously [24]. HttQP103 was subcloned to express non-fluorescent version of this Htt fragment. This latter construct was cotransfected at a 1∶1 ratio with the GFP-labeled HttQP25 to examine the mobility of HttQP25 in cells with inculsions. PolyQ19and polyQ81 were described previously [11]. Wild-type and mutant constructs of human SOD1cloned into the pBUD vector [25] were expressed as non-fluorescent proteins or else were cloned into pEGFP-C3 vector (Invitrogen). Human Hsp70 and human Hsp40 (Hdj1) were cloned into pFLAG-CMV2 (Kodak). To examine the effect of overexpression of Hsp70 or Hsp40 on the mobility of Htt fragments or SOD1 mutants, we routinely used 2 times more DNA of the heat shock protein than Htt or SOD1 DNA. As a control for inclusion formation, the Htt fragments were transfected with RFP-tagged clathrin light chain, instead of the molecular cochaperones.

### Cell culture and immunostaining

N2A and HeLa cells were obtained from ATCC. MN-1 cells are mouse motor neuron neuroblastoma cells [26]. Sn56 cells are mouse cholinergic septal neuronal cells [27]. Cell lines were grown in Dulbecco's Minimal Essential Medium (Invitrogen), except Minimal Essential Medium (Invitrogen) was used for N2A cells. Cells were transfected using Fugene (Roche) with 0.5 µg/ml DNA. Cells wereincubatedin 5 µg/mlLatrunculin A (Sigma-Aldrich) to depolymerizeactin or in 10 µg/mlnocodazole (Sigma-Aldrich) to depolymerize microtubules for 40 min at 37°C. To inhibit the proteasome, transfected cells were incubated overnight with 10 µmN-acetyl-leucyl-leucyl-norleucinal (ALLN) (Sigma) prior to lysis. To determine the efficiency of cotransfection of Htt and either Flag-tagged Hsp70 or Flag-tagged Hsp40, after fixing cells in 4% paraformaldehyde, they were immunostained with anti-FLAG antibody (#F1804, clone M2, Sigma), followed by donkey anti-mouse secondary antibody (#715-165-150, JacksonImmuno Research).

### FCS Measurement and Data Analysis

FCS measurements of GFP fusion proteins were carried out in live cells using the Zeiss Confocor system with a 40X water objective. Rhodamine 6G dye (Sigma-Aldrich) was routinely used to align the pinhole and to calibrate the dimensions of the instrument confocal volume. GFP was excited at the 488nm laser line of an argon laser using a 40X water immersion objective. FCS measurementswere performed by averaging a total of 30 1s consecutive measurements at room temperature using a minimum of 15 cells for each experimental condition to obtain a data set. The autocorrelationfunction, 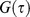, was globally analyzed to a 1-component model consisting of diffusion and triplet states as shown in equation 1:
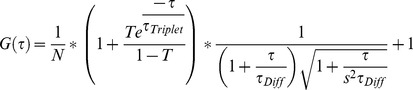
where *N* is the average number of molecules, *T* is the fraction of molecules in the triplet state, and *1/*τ*_T_* the corresponding triplet decay rate, τ_dif,_ is the diffusion time of single molecular species, 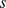 is the structural parameter, which is equivalent to ω*_z_*/ω*_xy_* where ω*_z_* and ω*_xy_* are the axial and equatorial radii of the confocal volume set up by the laser beam, respectively. The only parameter that was fixed was the structural parameter, which was set at a value of 5. The value was determined for this parameter when using the 488 nm laser line and the Confocor system [28].

In performing these measurements, the FCS program generated Chi square values for each autocorrelation curve that indicate the goodness of the fit and deviation plots that measure the difference between the fitted function and the experimental data. Diffusion times were only used from measurements with Chi square values of less than 5E-006 and deviation plots with no large systematic variations. The diffusion coefficients were calculated by averaging a minimum of 4 data sets each obtained from a different transfection set consisting of a minimum of 10 cells.

**Figure 1:**
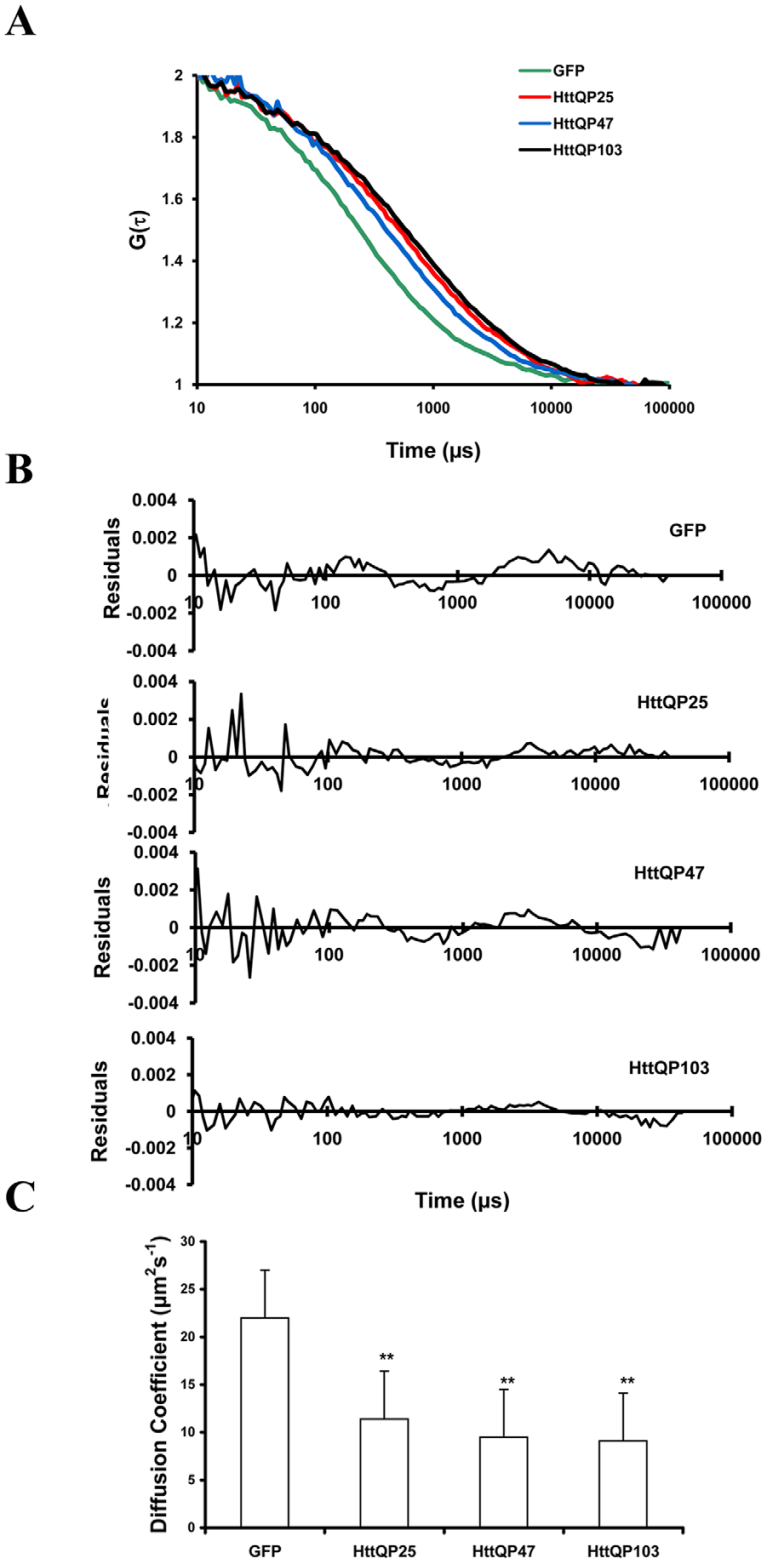
Diffusional mobility of Htt fragments in Sn56 cells. In A, the autocorrelation curves for cytosolic GFP, HttQP25, HttQP47, and HttQP103 were measured 72h after transfection. In cells expressing HttQP47 and HttQP103, the diffusional mobility was measured in cells without inclusions. Curves were normalized using the Confocor2 program. In B, the residuals for each of the autocorrelation curves shown in A. In C, diffusion coefficients of GFP, HttQP25, HttQP47, and HttQP103 were measured 72h after transfection. The diffusion coefficients and standard deviations were calculated by averaging a minimum of 4 data sets each obtained from a different transfection with each data set consisting of a minimum of 10 cells. **, *p*<0.005 determined by Student's *t*-test of significant difference between GFP and HttQPs diffusion coefficient.

**Table 1:** Diffusion coefficients measured in different cell lines.*

cell line	GFP	HttQP25	HttQP103
N2a	20.9±2.9	9.6±2.1	8.8±2.2
Hela	20.8±3.2	12.0±2.7	9.7±1.8

* Diffusion coefficients (given asµm^2^ s^−1^) were measured in cells without inclusions.

In addition to a 1-component fit, the autocorrelation curves obtained with the Htt fragments were also fitted to a 2-component fit. First, the autocorrelation curves were fitted with 2-components using the Confocor program to obtain average dwell times and fractional amounts of each of the components. However, this method yielded a diffusion time for the fast component that was 2- to 3- times faster than the diffusion time for GFP, indicating a lower molecular weight than GFP, which is not possible. We also fitted the Htt and SOD1 data to a 2-component fit with the diffusion time of one of the components fixed at the theoretical value calculated using the molecular weight of the GFP-labeled protein and the proportionality that diffusion scales with the cube-root of the molecular weight for globular proteins [29]. A diffusion time for the second component and the fraction of each component were then calculated using the Confocor program. The Chi square values for the 2-component fit were within 50% of the values obtained with a 1-component fit. In about half of the measurements, this 2-component fit showed a slightly better fit of the autocorrelation curves for Htt and SOD1 mutants. However, when a 2-component fit was used in fitting the data obtained for GFP alone, about a quarter of the fits likewise showed a 2-component fit gave a slightly better fit even though the cells contained only 1-component. This is consistent with the observation that the signal to noise ratios when using FCS to measure diffusional mobility is significantly worse in cells than in solution due to light scattering, autofluorescence, photobleaching of the dyes in restricted compartments, and cell damage from laser illumination [30]. Therefore, in analyzing our data, we only used the results of the 1-component fit.

**Figure 2:**
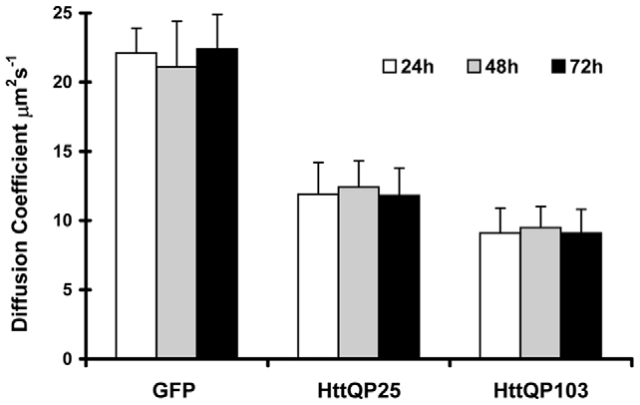
Time of expression does not affect the diffusion coefficients of GFP, HttQP25 and HttQP103. FCS measurements were made 24, 48, and 72h after transfecting the cells. Diffusion coefficients and standard deviations were calculated as described in Figure 1. Student's *t* test showed duration of expression had no significant effect on the measured diffusion coefficient.

### Western blots

After seeding cells in 6-well plate at 0.8×10^5^/well, cells were transfected with the different constructs using FuGene HD reagent (Roche). Two days post- transfection, cells were washed twice with phosphate buffer saline, scraped off the plate and then collected by gentle centrifugation. The pellet was resuspended in 200µl lysis buffer (50mM Tris-HCl, 0.1% nonidet P-40, 0.5% sodium deoxycholate, 150mM NaCl, 1mM EDTA, Protease inhibitor cocktail (Roche). After sonicating the cells on ice, the lysates were centrifuged to remove nuclei and large debris. Protein concentration of each lysate was determined using the Bradford reagent (Sigma) and then the same amount of protein was loaded in each lane. For SDS denatured gels, lysates were boiled in NuPage LDS sample buffer (Invitrogen) and then electrophoresed on NuPage 4–12% Bis-Tris gels (Invitrogen) using NuPage MOPS SDS running buffer (Invitrogen). For native gels, cell lysates mixed with Novex Native Tris-Glycine sample buffer (Invitrogen) were electrophoresed on 10% Tris-Glycine gels (Invitrogen) using Tris-Glycine Native running buffer (Invitrogen). Molecular weight markers from either Seeblue Plus2 (Invitrogen) or Rainbow Markers (GE Healthcare) for the SDS gels and the NativeMark Protein Standard (Invitrogen) for the native gel were used to calculate molecular weights. Gels were transferred to nitrocellulose and then immunoblotted with the following antibodies: anti-GFP polyclonal antibody (#Ab6556, Abcam), anti-HSP40 polyclonal antibody (Enzo ADI-SPA-400), anti-actin monoclonal (#AC-15, Abcam), anti-Hsp70 polyclonal antibody (#ADI-SPA-812, Enzo Life Sciences), anti-polyclonal SOD1 antibody (#ADI-SOD-100, Enzo Life Sciences), and anti-human SOD1 antibody (gift from Dr. Jeffrey Rothstein, John Hopkins University). Odyssey dye 800-conjugated donkey anti-rabbit (#926-32213, Licor) and Odyssey dye 700 donkey anti-mouse antibody (#926-32222, Licor) was used as secondary antibody. Odyssey scanner and software were used for detection and quantification of immunoblots.

**Figure 3:**
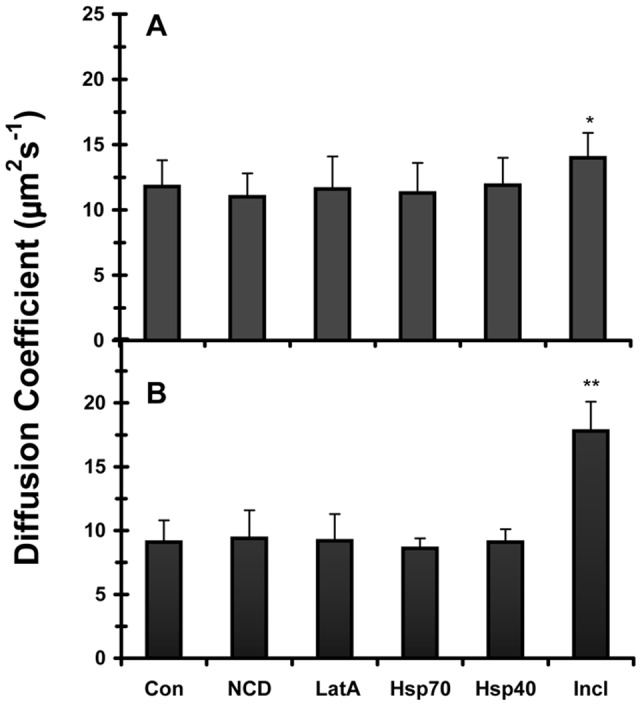
Diffusion coefficient of HttQP fragments measured under different conditions. The diffusion coefficients of cytosolic HttQP25 (panel A) and HttQP103 (panel B) were measured in control cells (Con), nocadozole-treated cells (NCD), Latrunculin A-treated cells (LatA), cells cotransfected with Hsp70 (Hsp70), cells cotransfected with Hsp40 (Hsp40), and cells with an inclusion (Incl). To obtain inclusions in HttQP25 expressing cells, cells were cotransfected with GFP-labeled HttQP25 and non-fluorescent HttQP103. FCS measurements were made 72h after transfection. Diffusion coefficients and standard deviations were calculated as described in Figure 1. *, *p*<0.05 and **, *p*<0.005 versus control cells by Student's *t*-test.

**Table 2:** Diffusion coefficients of different polyQproteins.^a^

Protein	Diffusion coefficient
GFP	22.2±3.2
HttQ25	14.4±2.1
HttQ103	11.6±2.3
PolyQ19	20.0±2.3
PolyQ81	14.6±2.6

a Diffusion coefficients (given asµm^2^ s^−1^)were measured in Sn56 cells without macro-aggregates.

### Filter Trap

MN-1 cells transfected with wild type or mutant pBUD-hSOD1 DNA were lysed 72 h after transfection using lysis buffer. Using a Bio-Rad dot blot device, the samples were filtered by gravity through 0.2 µm cellulose acetate membrane (Schleicher &Schuell) followed by two washes of Tris-buffered saline (TBS) with 0.5% Tween 20 (TTBS). The membranes were then incubated in 5% dry milk in TTBS at room temperature for 1 h, followed by an overnight incubation at 4^o^C with the anti-SOD1antibody in 5% dry milk in TTBS.

**Figure 4:**
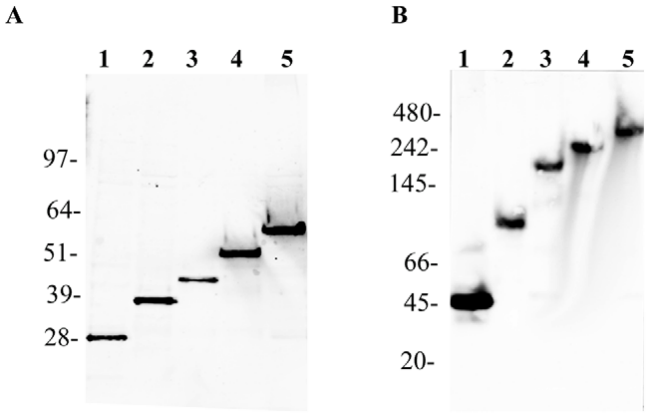
Western Blot analysis of lysates from cells transfected with Htt fragments. A. Western blot of GFP and different GFP-labeled Htt fragments run on a SDS polyacrylamide gel. B. Western blot of GFP and different GFP-labeled Htt fragmentsproteins run native gel. Lanes are as follows: GFP, lane 1; HttQ25, lane 2; HttQP25, lane 3, HttQ103, lane 4; and HttQP103, lane 5. After transferring the gels to nitrocellulose, anti-GFP antibody was used to detect the Htt fragments. The indicated molecular weights were determined using protein standards. The following are the calculated molecular weights of the different proteins based on their amino acid sequence: GFP, 30kDa. HttQ25, 36 kDa, HttQP25, 41 kDa, HttQ103, 48 kDa, HttQP103, 53 kDa.

## Results

### Diffusional mobility of polyQ proteins

FCS measures the fluctuation of the fluorescence intensity of molecules as they move through a small volume due to Brownian motion. When these fluctuations are analyzed using an autocorrelation function, the average diffusion time is obtained for the fluorescent molecule. Using this technique, the diffusion mobilities of GFP-tagged Htt fragments with varying lengths of polyQ repeats were first measured in the neuronal cell line Sn56. We expressed HttQP25, HttQP47, and HttQP103 fragments, containing the full-length exon 1 that includes the first 17 amino acids of Htt, followed by the polyQ repeat region, and then a polyproline stretch of amino acids fused to GFP. GFP alone was also transfected as a control. The GFP fluorescence was diffusely distributed throughout the cytoplasm in cells expressing HttQP25 (25 polyQs), whereas in cells expressing HttQP47 (47 polyQs) or HttQP103 (103 polyQs), there were both cells in which the GFP fluorescence appeared completely diffuse and cells containing a large visible aggregate (inclusion), many of which are probably aggresomes [31]. As expected, many more inclusions were present in cells transfected with the HttQP103 than in cells transfected with HttQP47. The diffusional mobilities of cytosolic Htt were measured in cells without inclusions throughout this entire study unless otherwise indicated.

**Figure 5:**
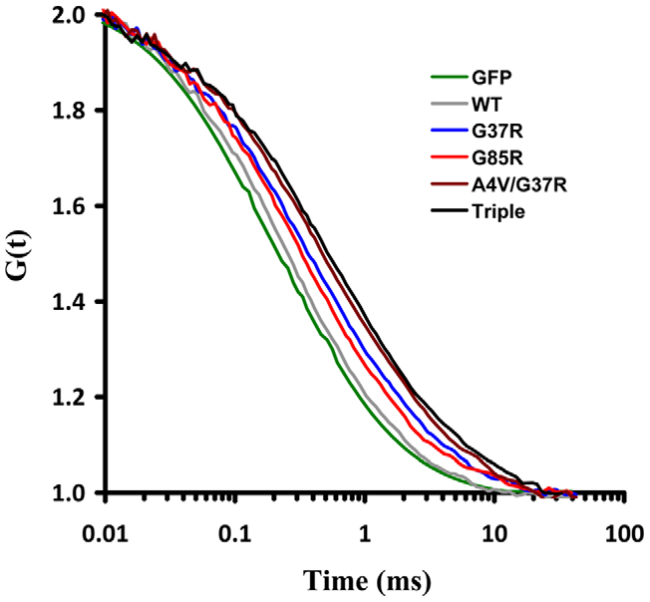
Diffusional mobility of wild-type SOD1 and SOD1 mutants in MN-1 cells. The autocorrelation curves for GFP, wild-type SOD1, and SOD1 mutants were measured 72h after transfection. Curves were normalized using the Confocor2 program.

**Figure 6:**
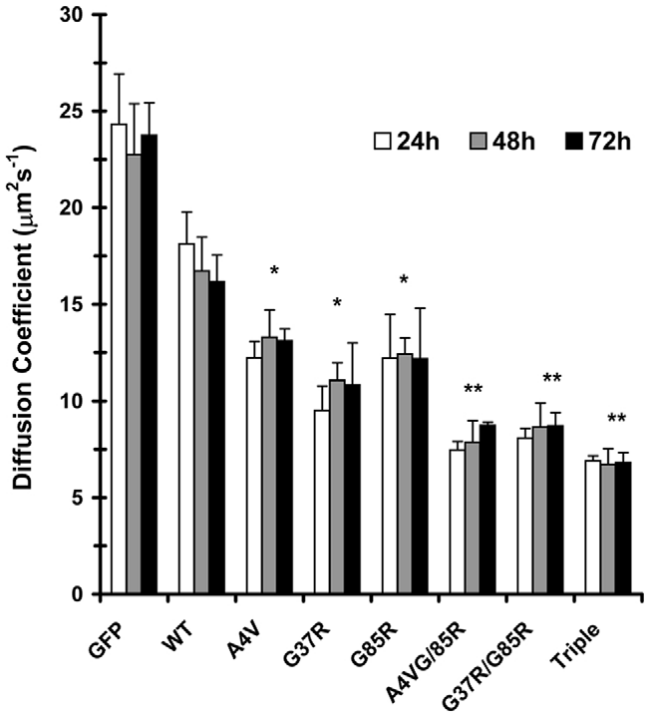
Time of expression does not affect the diffusion coefficients of wild-type and mutant SOD1 proteins. FCS measurements were made 24, 48, and 72h after transfection. Diffusion coefficients and standard deviations were calculated as described in Figure 1. **, *p*<0.005 of mutant SOD1 versus WT-SOD1 as determined by Student's *t*-test.

Fig. 1A and 1B shows typical autocorrelation curves for cytosolic GFP, HttQP25, HttQP47, and HttQP103, along with their respective deviation plots, measured 72h post-transfection. The autocorrelation curves were fitted using a 1-component fit since deviation plots showed that this provided a good fit to the data. From the diffusion time derived from the autocorrelation curve for cytosolic GFP, a diffusion coefficient was determined to be 22.2±3.2µm^2^ s^−1^, in good agreement with values from other laboratories [32], [33]. Compared to GFP, HttQP25, HttQP47, and HttQP103 diffused much more slowly in the cytosol with diffusion coefficients of 11.4±2.0, 9.5±1.8, and 9.1±1.9 µm^2^s^−1^, respectively (Fig. 1C). Interestingly, these values are significantly smaller than the predicted values of 19.8, 19.3, and 18.2 µm^2^s^−1^ for monomeric HttQP25, HttQP47, and HttQP103, respectively. These latter values were calculated using the measured diffusion coefficient of cytosolic GFP, the molecular weights of the Htt fragments, and the fact that the diffusion coefficient is proportional to the cube-root of the molecular weight for globular proteins [29]. This relationship was shown to be applicable to polyQ peptides by the Pappu laboratory [23]. These results show that neither the non-pathological nor the pathological HttQP fragments diffused as monomers in the cytosol. Based on their diffusion coefficients, their apparent molecular weights were about 5 to 8 fold greater than that of monomer. In addition to SN56 cells, similar diffusion coefficients for HttQP25 and HttQP103 were obtained in N2a and HeLa cells, suggesting that oligomerization is independent of cell type (Table 1).

**Figure 7:**
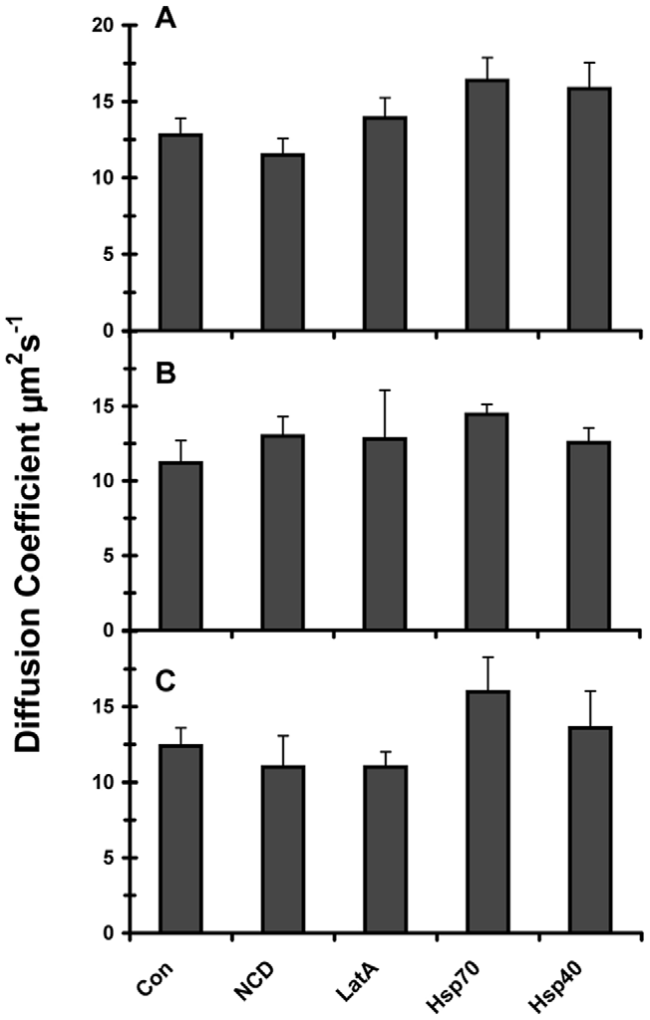
Diffusion coefficients of SOD1 with point mutations measured under different conditions. The diffusion coefficients of SOD1(A4V) (panel A), SOD1(G37R) (in panelB), and SOD1(G85R) (in panel C) were measured in control cells (Con), nocadozole-treated cells (NCD), Latrunculin A-treated cells (LatA), cells cotransfected with Hsp70 (Hsp70), and cells cotransfected with Hsp40 (Hsp40). FCS measurements were made 72h after transfection. For each expressed construct, Student's *t* test showed that the change in conditions had no significant effect on the measured diffusion coefficient.

To determine the factors that affect the diffusional mobility of the Htt fragments, FCS measurements of HttQP25 and HttQP103 were performed under different conditions in cells without inclusions unless otherwise indicated. First, we examined whether the time post-transfection affected their mobility. As shown in Fig. 2, the diffusion coefficients of HttQP25 and HttQP103 were not significantly different when measured at 24h, 48h, and 72h post-transfection in cells containing only diffusive fragments. Although the cytosolic mobility of HttQP103 was not affected over a 72h time period, there was a significant increase in the number of cells containing inclusions over this time period. Specifically, the percent of cells with inclusions increased from 15±4% at 24h post-transfection to 35±5% at 72h post-transfection.

**Figure 8:**
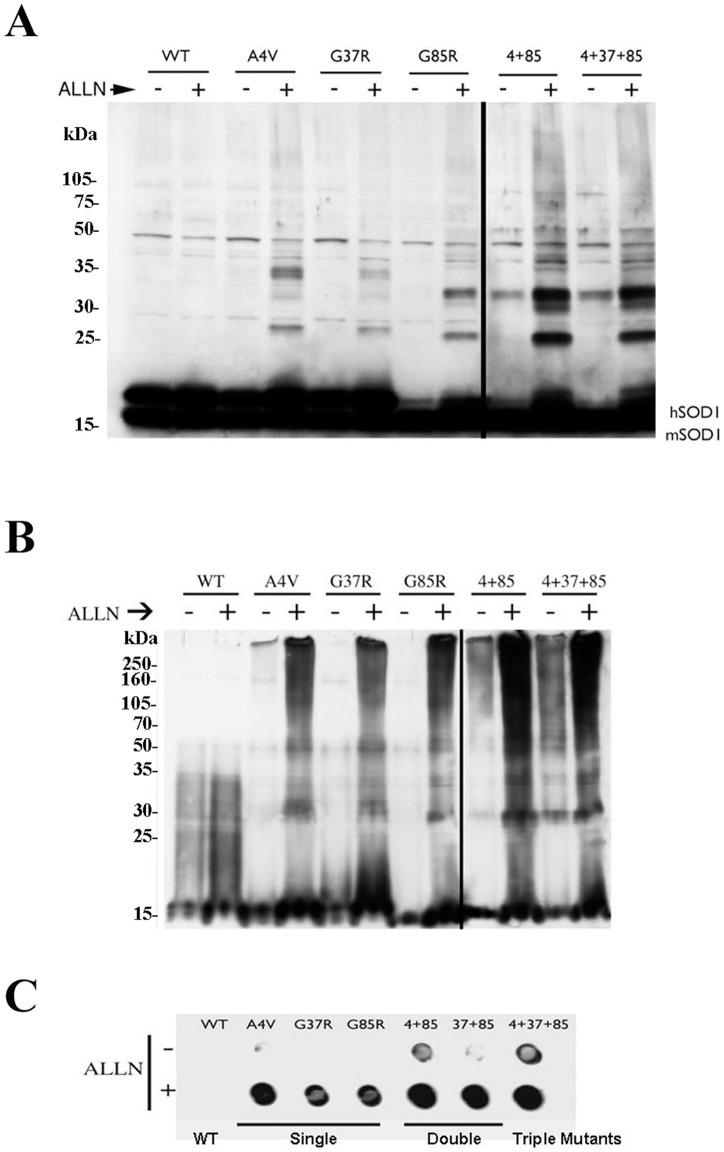
SOD1 with point mutations forms aggregates that are enhanced by proteasome inhibition. A. Western blot of WT and mutant SOD1 proteins run on a SDS gel. The antibody detects both the endogenous mouse SOD1 (mSOD1) and the expressed human SOD1 (hSOD1). B Western blot of WT and mutant SOD1 proteins run on a native gel. Antibody detects only the expressed human SOD1. C. Nitrocellulose filter assay of SOD1 to detect insoluble aggregates. As indicated, samples were incubated overnight with 10 µM proteasome inhibitor, (ALLN).

Next, we examined whether the slow diffusion of the cytosolic Htt fragments was due to their interaction with the cytoskeleton by disrupting the microtubules with nocodazole or depolymerizing fibrillar actin with Latrunculin A. As shown in Fig. 3, the diffusion coefficients for HttQP25 and HttQP103 were not significantly affected by depolymerization of these cytoskeletal components suggesting that the reduction in mobility is not caused by the interaction of the Htt fragments with the cytoskeleton. Rather, the slower diffusion is most likely due to oligomerization of the Htt fragments.

The diffusional mobility of the residual cytosolic Htt fragments was also measured in cells with an HttQP103 inclusion. In cells expressing GFP-HttQP103, this was done at 72h post-transfection to obtain many cells with inclusions. Interestingly, there was a marked increase in the diffusional mobility of the residual cytoplasmic HttQP103 in cells containing an inclusion (Fig. 3). The diffusion coefficient of the residual cytoplasmic HttQP103 is 17.8±3.3 µm^2^ s^−1^, which is within experimental error of the calculated value of 18.2 µm^2^ s^−1^ for monomeric HttQP103. These results show that after the majority of the cytoplasmic HttQP103 is sequestered into an inclusion, the residual cytosolic HttQP103 has a diffusion coefficient consistent with it being monomeric. Although monomer, itself, has been reported to be toxic [9], [10], presumablythe monomer left in the cytosol is non-pathologic given that the rate of cell death is less dependent on cytosolic Htt concentration in cells containing an inclusion than in cells with only diffuse fragments [9]. Importantly, the fact the HttQP103 is monomeric in cells with an inclusion makes it highly unlikely that the increase in the apparent molecular weight of HttQP103 in cells without inclusions is due to their interaction with other proteins; there is no reason why such interactions would not also occur in cells with inclusions.

To test whether the presence of an inclusion would similarly increase the diffusional mobility of HttQP25, cells were cotransfected with GFP-labeled HttQP25 and non-fluorescent HttQP103 since cells expressing only HttQP25 don't have inclusions even when expressed at very high levels. Many of the cotransfected cells contained a fluorescent inclusion, which showed that HttQP25 coassembled with HttQP103 into inclusions, as previously reported [24]. Measurement of the diffusional mobility of the residual cytosolic HttQP25 in cells with inclusions yielded a diffusion coefficient of 14.0±2.0 µm^2^ s^−1^ (Fig. 3). This value is significantly larger than 11.8±2.0 µm^2^ s^−1^, the measured diffusion coefficient of cytosolic HttQP25 in cells expressing only HttQP25. However, it is still significantly smaller than the calculated value of 19.8 µm^2^s^−1^ predicted for monomeric HttQP25. These results show that HttQP103 inclusions take up both oligomeric HttQP103 and oligomeric HttQP25 from the cytosol, but they take up much more of the former than the latter.

Finally, we investigated whether the molecular chaperones, Hsp70 and Hsp40 (Hdj1), increased the diffusional mobility of HttQP103 since overexpression of molecular chaperones has been shown to reduce formation of polyQ inclusions [34], [35], [36]. In agreement with these studies, cotransfection with HttQP103 and either Hsp70 or Hsp40 caused a 50% reduction in transfected cells with inclusions. Consistent with this reduction, immunostaining of the molecular chaperones showed that 85–90% of the cells with GFP fluorescence also expressed either Hsp70 or Hsp40. Immunostaining showed a diffuse cytoplasmic localization of these chaperones, while in cells with inclusions, in addition to the cytosolic staining, thesechaperones accumulated around the inclusion (Fig. S1), as shown previously [37], [38]. As for the level of expression of the Hsp70 and Hsp40 in the transfected cells, Western blot analysis showed that these molecular chaperones are 10-fold more abundant than the endogenous level of these proteins without correcting for the ∼50% transfection efficiency (Fig. S2).

Previous studies have shown that the amino acid composition of the polyQ fragments profoundly affects their aggregation and cytotoxicity. For example, Htt fragments of different lengths, but with the identical polyQ expanded repeat regions, have very different aggregation properties and cytotoxicity in tissue culture cells [39]. In vitro studies have shown that the N-terminal flanking region enhanced the formation of polyQ inclusions [40], whereas a C-terminal polyprolineflanking region caused a reduction in the aggregation rate and the stability of the polyQ inclusions [41]. To determine the effect of a flanking region on formation of oligomers, we measured the diffusional mobility of cytosolic HttQ25 and HttQ103 fragments. These Htt fragments are identical to the ones used above except they do not have the C-terminal polyproline flanking region. FCS measurements yielded values of 14.4±2.1 and 11.6±2.3 µm^2^ s^−1^ for the diffusion coefficients of cytoplasmic HttQ25 and HttQ103, respectively (Table 2). These values are significantly smaller than the calculated values of 19.8 and 18.8 µm^2^ s^−1^for monomeric HttQ25 and HttQ103, respectively. Therefore, these results show again that both the non-pathological and pathological Htt fragments form oligomers in the cytosol. In addition, these results show that the diffusion coefficients of HttQ25 and HttQ103 are significantly larger than the diffusion coefficients of HttQP25 and HttQP103, respectively. This difference in diffusional mobilites between the HttpolyQ and HttpolyQP fragments may be due to differences in the shape of the oligomers.

Lysates from cells transfected with either GFP or GFP-labeled Htt fragments were run both on SDS gels and native gels. As shown in Fig. 4A, the mobilities of the different Htt fragments, HttQ25, HttQP25, HttQ103, and HttQP103, on SDS gels are consistent with their monomeric molecular weights. However, when these same lysates were run on native gels, these proteins had slower mobility than expected based on their molecular weights (Fig. 4B). GFP migrated at a molecular weight of about 45kDa, whereas all of the Htt fragments showed a much greater retardation in their mobility. Based on the molecular weight standard, the HttQP25 and HttQP103 fragments are migrating 5–6 fold slower than expected from their molecular weights, while the HttQ25 and HttQ103 are migrating 3–5 fold slower than expected based on their molecular weights. These results are consistent with the FCS data that showed the HttQP fragments migrate significantly slower than the HttQ fragments.

FCS measurements were also performed on two other polyQ fragments, polyQ19 and polyQ81, composed of a N-terminal sequence of 9 amino acids of which 5 are histidine residues, followed by the polyQ repeat region and then 8 amino acids from the atrophin-1 protein that finally links to the C-terminal GFP. Takashi et al [11] examined these polyQ fragments in FCS measurements using cells lysates. In their experiments, oligomers were found in lysates from cells expressing polyQ81, but not in lysates from cells expressing polyQ19. Our FCS measurements showed that both of these cytosolic polyQ fragments had faster diffusional mobility than the comparable Htt fragments (see Table 2). From the autocorrelation curve, the diffusion coefficient of cytosolic polyQ19 was determined to be 20.0±2.3 µm^2^ s^−1^, within experimental error of the calculated diffusion coefficient of 20.6 µm^2^ s^−1^ for monomeric polyQ19. Therefore, in agreement with the Takashi et al study [11], our FCS measurements showed that cytosolic polyQ19 occurs as monomer in the cytosol, unlike the cytosolic HttQ25 and HttQP25 fragments. Even though polyQ19 is monomeric, it is sequestered in inclusions when cotransfected with either non-color HttQP103 or HttQ103, as shown previously [38].

As for polyQ81, FCS measurements performed in cells without inclusions yielded a value of 14.6±2.6 µm^2^s^−1^ for its diffusion coefficient, which is significantly smaller than 18.8 µm^2^s^−1^, the calculated value for monomeric polyQ81. This shows that polyQ81 is forming oligomers in the cytosol unlike polyQ19. However, when the mobility of polyQ81 was measured in cells with inclusions, the diffusion coefficient of the cytosolic polyQ81was increased to 18.1±3.1µm^2^s^−1^, which shows that the residual cytosolic protein is monomeric. Therefore, just as we observed with HttQP103, the polyQ81 oligomers in the cytosol appear to be sequestered in the inclusion, leaving behind monomeric polyQ81in the cytosol. In addition, the fact that these polyQ fragments have faster mobility than the comparable size Htt fragments is consistent with the recent observation that the 17 amino acid N-terminal residues of Htt greatly enhanced its aggregation into globular oligomers [40].

### Diffusional mobility of wild-type and mutant SOD1 proteins

Mutations in SOD1 cause a dominantly inherited form of amyotrophic lateral sclerosis (FALS). Pathological examination of motor neurons from patients with SOD1-related ALS reveals cytoplasmic aggregates of SOD1, a feature that is recapitulated in cell culture expression of mutant SOD1. Unlike huntingtin, however, the aggregation of SOD1 is caused by point mutations and so far, more than 100 disease-causing mutations have been identified. We studied three of the naturally occurring single point mutants, A4V, G37R, and G85R, as well as two double point mutants, A4V/G37R and G37R/G85R, and a triple point mutant, A4V/G37R/G85R. Both the wild-type and mutant SOD1 proteins were expressed as GFP fusion proteins in MN-1 cells. In general, the fluorescence was diffusely distributed throughout the cytoplasm, but there was an occasional visible aggregate in cells expressing a mutant construct of SOD1. Our observations are consistent with previous studies in which less than 5% of the cells expressing mutant SOD1 had visible aggregates, as well as with sedimentation studies of cell lysates, which showed that most of the mutant SOD1remained in the supernatant [25], [42], [43].

Fig. 5 shows typical autocorrelation curves for GFP, wild-type SOD1 and the following SOD1 mutants: SOD1(G37R), SOD1(G85R), SOD1(A4V/G37R), and SOD1(A4V/G37R/G85R). Although not shown for the sake of clarity, the autocorrelation curves for SOD1(A4V) and SOD1(G37R/G38R) overlapped with the curves for SOD1(G85R) and SOD1(A4V/G37R), respectively These curves illustrate that mutations of a single amino acid residue caused a reduction in the mobility of SOD1 and increasing the number of point mutations caused a further reduction in mobility. Turning first to the wild-type protein, its diffusion coefficient was determined to be 16.7±1.4 µm^2^s^−1^, about two-thirds of the value measured for GFP (23.7±2.1 µm ^2^s^−1^). This measured value for wild-type SOD1 is in excellent agreement with the calculated value of 16.3 µm ^2^s^−1^ based on two 16-kDa subunits of SOD1 each fused to a GFP molecule. As for the single point mutants, regardless of the time of expression, they showed a 25%–40% reduction in their diffusion coefficients relative to that obtained for wild-type SOD1 (Fig. 6). Finally, the double and triple point mutants showed about a 50% reduction in their diffusion coefficients relative to the wild-type value (Fig. 6). The fact that the diffusion coefficients of the mutant SOD1 proteins are decreased relative to the wild-type SOD1 indicates that the apparent molecular weights of the mutants are greater than that of the wild-type protein. Based on our FCS measurements, SOD1(A4V) and SOD1(G85R) form oligomers composed of 2–3 homodimers, while the SOD1(G37R) mutant is composed of about 5 homodimers. As for the double and triple point mutants, they appear to form oligomers composed of 8–10 homodimers.

In regard to the effect of the experimental conditions, we found that neither the addition of nocodazole to depolymerize the microtubules nor Latrunculin A to depolymerize the actin significantly affected the diffusion coefficients of the single point mutants of SOD1 (Fig. 7). Likewise, their diffusion coefficients were not affected by overexpression of Hsp70 or Hsp40. In summary, FCS measurements show that the presence of point mutations in SOD1 caused a reduction in their mobility, which indicates an increase in their apparent molecular weights consistent with the formation of small oligomers.

Since FCS microscopy was performed using GFP fusion proteins of SOD1, we wanted to confirm that the GFP label was not causing the oligomerization of the SOD1 mutants. Therefore, we expressed these same mutants of SOD1 without the GFP label. To examine their aggregation state, we made cell lysates from the transfected MN-1 cells and then ran the lysates on SDS and native polyacrylamide gels, followed by Western blot analysis. In agreement with Krishnan et al [25], the mutant SOD1 proteins ran as higher molecular weight complexes both on denaturing and native gels (Fig. 8). Furthermore, as shown both by the gels and the dot blot in Fig. 8, oligomerization of the SOD1 mutants became more pronounced following overnight incubation with the proteasome inhibitor, ALLN, as observed previously [42]. In contrast, wild-type SOD1 did not polymerize even in the presence of proteasome inhibitor, in agreement with previous reports [25], [42]. Therefore, these data further support the view that point mutation of SOD1 results in the formation of small cytosolic oligomers.

## Discussion

Our results indicate that both non-pathological and pathological Htt fragments occur as oligomers in the cytosol. Interestingly, even though HttQP103 and polyQ81 fragments were oligomeric in cells without inclusions, their diffusional mobility indicated that they were monomericin cells containing a large inclusion. Thefact that the residual HttQP103 and polyQ81 have the diffusional mobility of monomer in cells with inclusions strongly suggests that, when these fragments have a slower mobility, it is due to their oligomerization rather than to their interaction with other proteins. If interactions with other proteins were indeed responsible for their slower mobility, presumably, these interactions would also occur in cells with inclusions. In addition, unlike HttQP25, polyQ19 had the mobility of monomer even though both of these fragments would be expected to interact similarly with other cytosolic proteins. Therefore, our data suggest that, like HttQP103 and polyQ81, HttQP25 is also oligomeric.

Our results are consistent with several studies that recently observed soluble Htt oligomers in the cytosol, using techniques including fluorescent microscopy, atomic force microscopy, agarose gel electrophoresis, and sedimentation velocity measurements [13], [40], [44], [45], [46], [47]. In determining the diffusional mobility, and in turn, the size of the oligomers, the autocorrelation curves were fitted using a 1-component fit. Thisyielded molecular weights for the Htt fragments consistent with the values determined using native gel electrophoresis. Specifically, the different Htt fragments ran as one major band with molecular weights significantly higher than that of monomer. However, we cannot rule out that this agreement is coincidental because on native gels, the proteins runanomalously due to charge and conformation. Therefore, the autocorrelation curves were also fitted using a 2-component fitalong with the assumption that one of the components was monomersince a 2-component fit was not compatible with the data without this assumption (see Materials and Methods). Although this fit suggests that oligomer representsonly about 25% of the total Htt fragment pool with the rest being monomer, but the size of the oligomer is approximately 8-fold larger than the size derived from the autocorrelation curve using a 1-component fit. Therefore, regardless of whether a 1- or 2- component fit is used, these results show that a significant fraction of the Htt forms large oligomers in the cytosol.

Our FCS measurements showed that the diffusional mobilities of the HttQP25 and HttQP103 oligomers were not dependent onthe length of time following transfection or the presence of the molecular chaperones, Hsp70 and Hsp40. However, even though these factors did not affect oligomer formation, they did affect the formation of visible polyQ inclusions, in agreement with previous studies [34], [35]. Specifically, increasing time favored the formation of visible inclusions, whereas expression of molecular chaperones reduced visible inclusion formation. It seems paradoxical that the chaperones did not reduce the size of the oligomers, which have been postulated to be pathological, but did reduce the formation of the inclusions, which have been postulated to be neuroprotective [7], [8]. One possibility, which was proposed to explain the in vitro atomic force microscopic structures obtained with Htt fragments in the presence and absence of molecular chaperones [48], is that Hsp70 and Hsp40prevent toxic off-pathway oligomeric assemblies and by binding to the oligomers, neutralize their toxicity. On this basis, inclusions would only form when the protection provided by the binding of the chaperones to the oligomers becomes overwhelmed so that the more chaperones that are present, the less the chance that inclusions would form.

Until recently, the formation of inclusions has been considered to be totally dependent on the length of the polyQ region. This paradigm has been challenged by a recent study from the Kopito laboratory [49]. They found that, in cells stably expressing HttQ25, the HttQ25 initially formed inclusions due to the presence of HttQ71, and then continued to form visible aggregates even after cell division had completely diluted out the HttQ71. This led them to propose that Htt aggregation can be transmitted in a prion-like manner. However, based on our data that HttQ25 already forms soluble oligomers in cells in the absence of HttQ71, the results from the Kopito laboratory would indicate that the transient presence of HttQ71 permanently alters the conformation of the HttQ25 oligomers so that they now form visible aggregates not only in the presence of HttQ71, but even after it is diluted out.

As for SOD1, only the mobility of wild-type SOD1 was consistent with it forming a homodimer of 16-kDa subunits. The three SOD1 proteins with single point mutations showedsimilar reductions in mobility even though their mutations affected different functional domains of the protein including the dimer interface (A4V), the beta sheets (G35R), and the active site (G85R). All three mutants have been shown to reduce the stability of SOD1, but only G85R causes a loss of enzymatic activity [50]. When these point mutants were combined to make double and triple point mutants, this caused a significant reduction in their diffusional mobilitiescompared to the single point mutants of SOD1. The decrease in the mobility of the SOD1 mutants indicates an increase in their apparent molecular weights, suggesting that the mutants are forming oligomers in the cytosol. As with Htt data, the autocorrelation curves were fitted to a 1-component fit, but here again, the results are compatible with 2-components consisting of dimers and oligomers.

Analysis of immunoprecipitates of cell lysates showed that SOD1 mutants, but not wild-type protein, bind various heat shock proteins, including Hsp70, Hsp40 and Hsp25 [51], [52]. In addition to binding to SOD1 mutants, these heat shock proteins have been shown to reduce aggregate formation and toxicity in neuronal cultures [52], [53], [54]. However, we did not find that markedly overexpressing Hsp70 and Hsp40 affected the diffusional mobility of the point mutants. Therefore, it seems very unlikely that the slower mobility of the mutants is mainly due to their binding the heat shock proteins. Instead, the results are consistent with the presence of oligomers in the cytosol. This conclusion is supported by the study of Krishnan et al. [25], as well as our own experiments, measuring the aggregation properties of SOD1 mutants with no GFP label. Western blot analysis of cell lysates showed that the SOD1 mutants, but not wild-type SOD1, formed aggregates. In addition oligomers of mutant SOD1(G85R) have been shown to be present in spinal cord extracts from transgenic mice expressing this SOD1 mutant [55].

Interestingly, although all three single point mutants of SOD1 appeared to form oligomers of similar size in the cytosol, there appears to be no direct relationship between the size of the oligomers and their cytotoxic effect in FALS patients. The survival time of FALS patients with the G85R and G37R point mutations of SOD1 are 6 and 18 years, respectively, whereas FALS patients with the A4V point mutation live about one year after onset of the disease [56]. It has recently been proposed that aggregation of mutant SOD1 protein is a major factor in disease progression based on biophysical techniques to calculate aggregation rates [57]. However, results from a recent study in which a large number of point mutants of SOD1 were expressed in cultured cells showed that aggregation and cytotoxicity may have a more complex relationship [58]. By measuring the extent offormation of detergent insoluble aggregates, it was found that patients with mutants having a low to moderate tendency to aggregate, there was a wide variation in the duration of the disease, whereas there generally was fast progression of the disease in patients with mutants having a high tendency to aggregate. Interestingly, the A4V and G85R mutants showed more detergent insoluble aggregation at 24h post-transfection than the G37R mutant, although they showed similar aggregation at 48h post-transfection [58]. The complexity of disease progression is further illustrated by the observation that the amount of native SOD1 in combination with mutant SOD1 affected disease progression in mouse models of ALS [59], [60]. Therefore, the aggregation of mutant SOD1 into soluble oligomers and detergent insoluble aggregates appears to be just one factor that affects the rate of FALS disease progression.

In conclusion, the results we obtained using FCS microscopy have shown that both non-pathological and pathological Htt fragments are present as oligomers in the cytosol. Likewise, SOD1 with point mutations appear to form oligomers in the cells; in fact, this is the first time that SOD1 oligomers have been detected in tissue culture cells. Most importantly, our results for both the Htt fragments and the SOD1 mutants indicate that cytotoxicity is not just due to the presence of oligomers or their size, but mainly depends on the specific properties of the oligomers.

## Supporting Information

Figure S1 **Localization of Hsp70 and Hsp40 in cells cotransfected with HttQP103.** Cells cotransfected with GFP-HttQP103 and either Hsp70 (upper panel) or Hsp40 (lower panels) were imaged by confocal microscopy. Cells were immunostained using an anti-Flag antibody to detect Hsp70 and Hsp40.
(TIF)

Figure S2 **Level of overexpressed Hsp70 and Hsp40 in cells cotransfected with Htt fragments.** In A, SDS gel showing expression of Hsp70 in cells transfected with Htt fragments alone or cotransfectected with Htt fragments and Hsp70. In B, SDS gel showing expression of Hsp40 in cells transfected with Htt fragments alone or cotransfectected with Htt fragments and Hsp40. Cells were cotransfected with only Htt fragments or Htt fragments and either Flag-tagged Hsp70 or Flag-tagged Hsp40 at a ratio of 1∶2. Western blot were prepared from cell lysates 48h after transfection. The immunoblots were probed with anti-GFP antibody to detect Htt, either anti-Hsp70 or anti-Hsp40 antibody, and anti-actin antibody as an internal loading protein control. All antibodies are described in the Materials and Methods.
(TIF)
