# Supplementary figures and images for: Huntingtin Fragments and SOD1 Mutants Form Soluble Oligomers in the Cell
Source: PLoS One. 2012 Jun 29;7(6):e40329. doi: 10.1371/journal.pone.0040329 (PMC3386994; doi:10.1371/journal.pone.0040329)

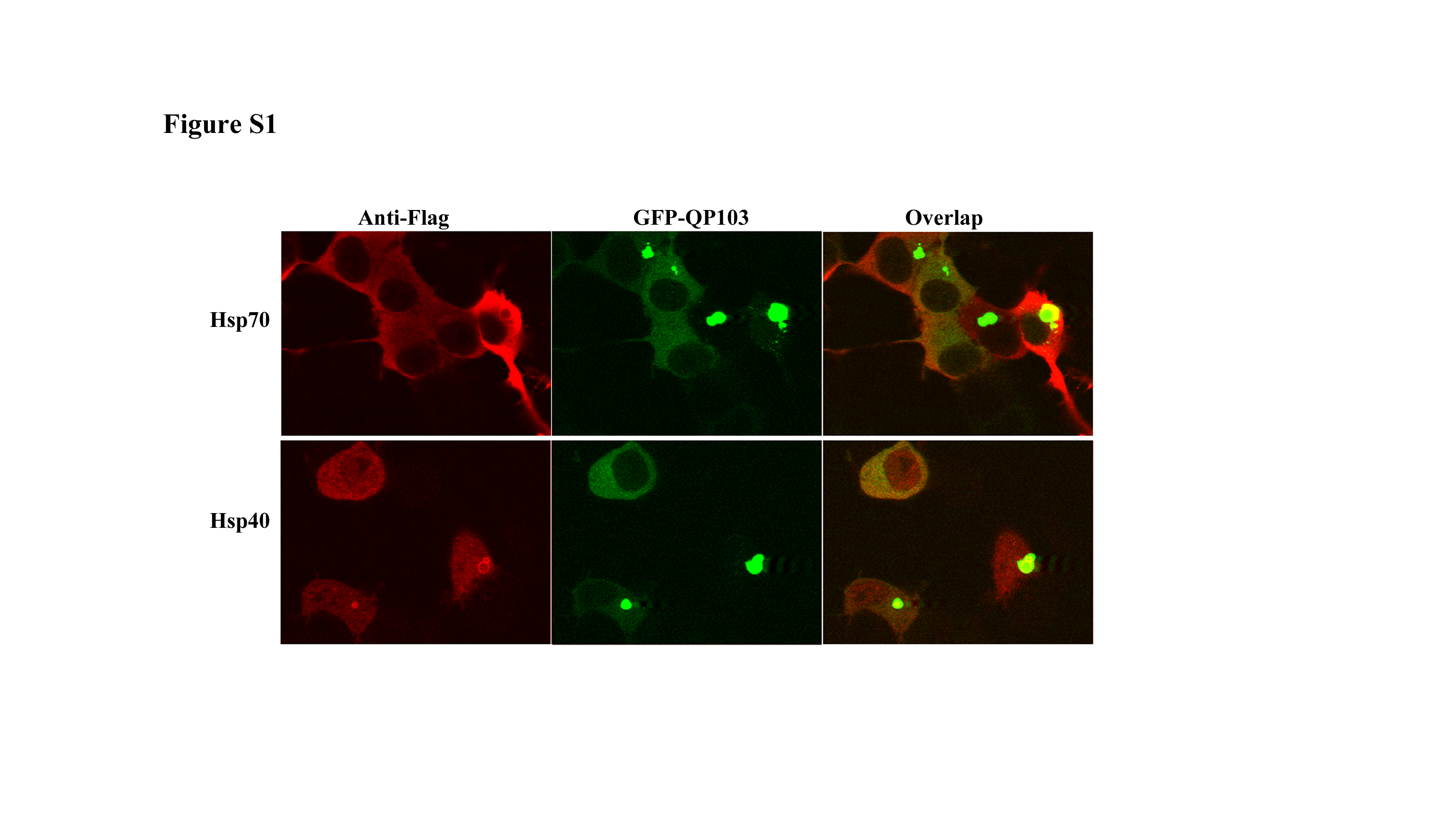

Supplement: Figure S1 — Localization of Hsp70 and Hsp40 in cells cotransfected with HttQP103. Cells cotransfected with GFP-HttQP103 and either Hsp70 (upper panel) or Hsp40 (lower panels) were imaged by confocal microscopy. Cells were immunostained using an anti-Flag antibody to detect Hsp70 and Hsp40. (TIF) [file pone.0040329.s001.tif]

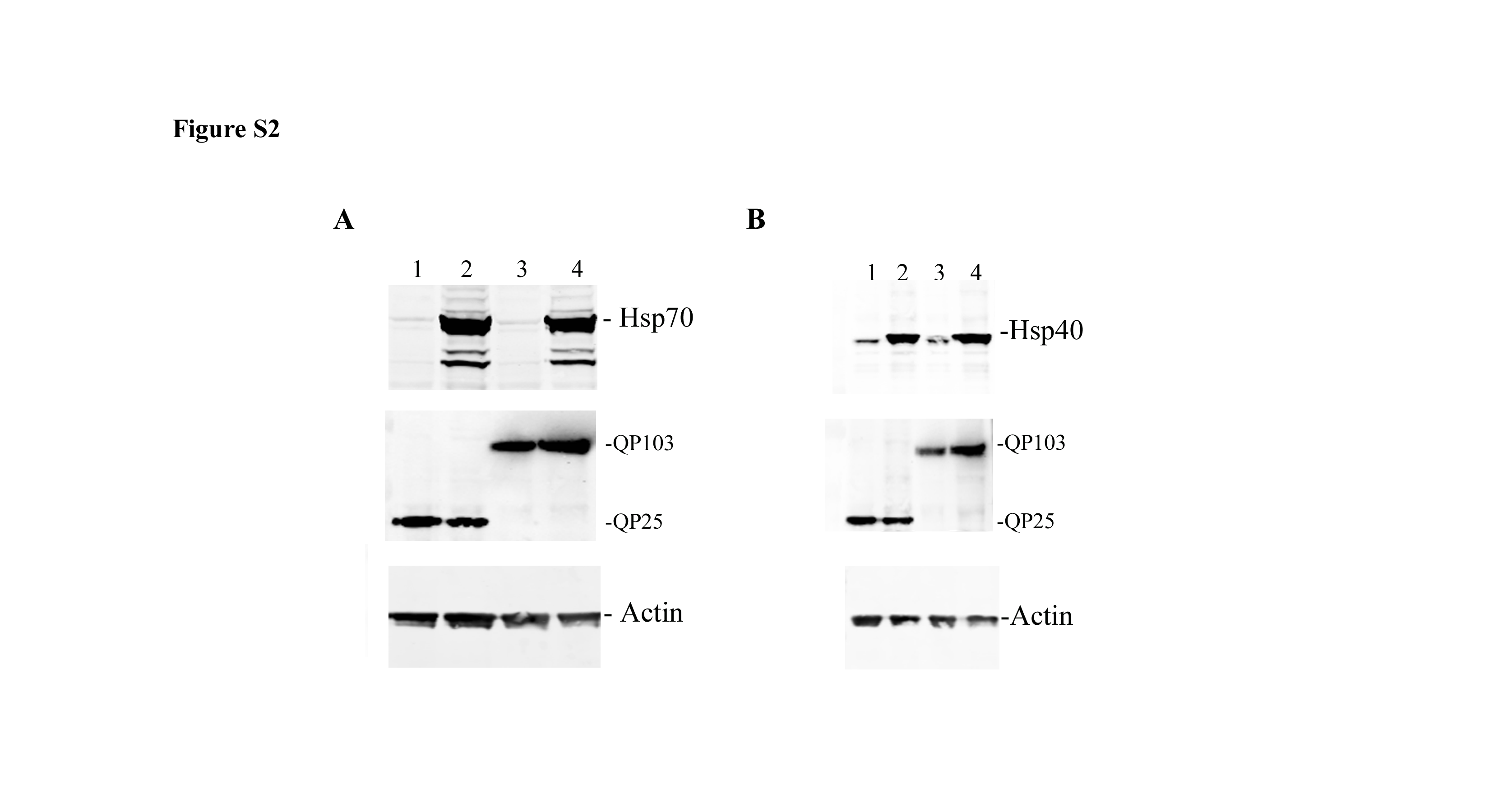

Supplement: Figure S2 — Level of overexpressed Hsp70 and Hsp40 in cells cotransfected with Htt fragments. In A, SDS gel showing expression of Hsp70 in cells transfected with Htt fragments alone or cotransfectected with Htt fragments and Hsp70. In B, SDS gel showing expression of Hsp40 in cells transfected with Htt fragments alone or cotransfectected with Htt fragments and Hsp40. Cells were cotransfected with only Htt fragments or Htt fragments and either Flag-tagged Hsp70 or Flag-tagged Hsp40 at a ratio of 1∶2. Western blot were prepared from cell lysates 48h after transfection. The immunoblots were probed with anti-GFP antibody to detect Htt, either anti-Hsp70 or anti-Hsp40 antibody, and anti-actin antibody as an internal loading protein control. All antibodies are described in the Materials and Methods. (TIF) [file pone.0040329.s002.tif]
